# Reference Ranges for Left Ventricular Curvedness and Curvedness-Based Functional Indices Using Cardiovascular Magnetic Resonance in Healthy Asian Subjects

**DOI:** 10.1038/s41598-020-65153-3

**Published:** 2020-05-21

**Authors:** Xiaodan Zhao, Soo-Kng Teo, Liang Zhong, Shuang Leng, Jun-Mei Zhang, Ris Low, John Allen, Angela S. Koh, Yi Su, Ru-San Tan

**Affiliations:** 1 National Heart Research Institute Singapore, National Heart Centre Singapore, 5 Hospital Drive, Singapore, 169609 Singapore; 2 Institute of High Performance Computing, Agency for Science, Technology and Research (A*STAR), 1 Fusionopolis Way, #16-16 Connexis, Singapore, 138632 Singapore; 3 Duke-NUS Medical School, 8 College Road, Singapore, 169857 Singapore

**Keywords:** Translational research, Cardiovascular diseases, Image processing, Ageing, Biomedical engineering

## Abstract

Curvature-based three-dimensional cardiovascular magnetic resonance (CMR) allows regional function characterization without an external spatial frame of reference. However, introduction of this modality into clinical practice is hampered by lack of reference values. We aim to establish normal ranges for 3D left ventricular (LV) regional parameters in relation to age and gender for 171 healthy subjects. LV geometrical reconstruction and automatic calculation of regional parameters were implemented by in-house software (CardioWerkz) using stacks of short-axis cine slices. Parameter normal ranges were stratified by gender and age categories (≤44, 45–64, 65–74 and 75–84 years). Our software had excellent intra- and inter-observer agreement. Ageing was significantly associated with increases in end-systolic (ES) curvedness (*C*_*ES*_) and area strain (*AS*) with higher rates of increase in males, end-diastolic (ED) and ES wall thickness (*WT*_*ED*_, *WT*_*ES*_) with higher rates of increase in females, and reductions in ED and ES wall stress indices (*σ*_*i,ED*_) with higher rates of increase in females. Females exhibited greater ED curvedness, *C*_*ES*_, *σ*_*i,ED*_ and *AS* than males, but smaller *WT*_*ED*_ and *WT*_*ES*_. Age × gender interaction was not observed for any parameter. This study establishes age and gender specific reference values for 3D LV regional parameters using CMR without additional image acquisition.

## Introduction

Structural and functional changes occur in the left ventricle (LV) during disease processes and over the course of chronological ageing. Cardiac magnetic resonance (CMR), specifically Simpson’s method employing phasic endocardial border contouring of a stack of parallel LV short-axis two-dimensional (2D) cine CMR images, is long regarded as the gold standard for quantification and assessment of global LV function and volumes due to its superiority over other modalities like echocardiography in terms of imaging quality, accuracy and reproducibility. Current CMR feature tracking (FT) techniques allow characterization of LV regional —and when aggregated, global— function, but are ultimately still based on tracking of 2D cine CMR images, commonly the LV basal, mid-cavity and apical short-axis slices^1^. The spatially spread-out 2D image slices constitute a sparse sampling of LV geometry and can neither capture changes in LV deformation in their entirety nor account for through-plane displacements. To comprehensively quantify LV geometry and longitudinal changes associated with remodeling in disease or with ageing, three-dimensional (3D) techniques potentially offer greater sensitivity and precision. This will be particularly helpful for the challenging task of quantitative characterization of LV geometry alterations among non-diseased healthy male and female subjects of various age groups, who do not exhibit gross LV remodeling.

In our previous studies, regional ventricular curvedness, thickness, curvature-based wall stress index and area strain were automatically evaluated from 3D reconstructed LV geometry in diverse of LV pathologies such as ischemic dilated cardiomyopathy^2^, heart failure (HF)^3^, myocardial infarction^4,5^ and hypertrophic cardiomyopathy^6^, as well as predominant right ventricular pathology such as repaired tetralogy of Fallot^7^. In this study, we hypothesize that our 3D technique can be applied in healthy cohorts to characterize gender- and age-related differences in LV geometry. Our results will provide reference values for the 3D characterization technique which will facilitate future clinical applications in healthy cohorts for both genders across various age groups.

## Results

### Baseline demographics of study population

The analysis cohort comprised 171 subjects without prior history of cardiovascular disease, hypertension, dyslipidemia or diabetes, and with LV ejection fraction (EF) ≥ 50% on CMR. Demographic data were collected at the time of imaging. In the evaluation of age-related characteristics, subjects were stratified into the four age (M/F) groups based on definitions of young, mid-age, young-old and medium-old by the Department of Statistics Singapore^8^: ≤44 years (30/36), 45–64 years (16/22), 65–74 years (30/23) and 75–84 years (7/7).

Table 1 summarizes the baseline demographics, clinical and CMR-derived LV parameters of study subjects (Appendix Table 1 tabulates the comparison of same parameters based on age groups of 10 years: 20–29 years; 30–39 years; 40–49 years; 50–59 years; 60–69 years; ≥70 years). The mean age was 52  ±  19 years; 83 (49%) were male. Mean LV end-diastolic volume (EDV) index, end-systolic volume (ESV) index, LV EF and mass index were within normal limits^9^. As expected, systolic blood pressure was significantly higher in the 65–74 years and 75–84 years age categories than the ≤44 years and 45–64 years categories. LV EDV index and ESV index were significantly smaller in the 75–84 years age group than the other three groups (all *P* < 0.001). There was a significant trend towards increased LV mass-to-volume ratio with increased age (*P* = 0.017). Sex-specific comparison of demographics are tabulated in Table 2. Systolic and diastolic blood pressure, LV EDV, ESV, LV mass indices, and LV mass-to-volume ratio were greater in males than females (all *P* < 0.05). Heart rate and LV EF did not differ significantly between males and females (*P* = 0.423 and *P* = 0.414, respectively).

**Table 1:** Baseline demographics, clinical and left ventricular parameters for all subjects and by age groups.

Parameters	Total (n = 171)	≤44 (Young) (n = 66)	45–64 (Mid-age) (n = 38)	65–74 (Young-Old) (n = 53)	75–84 (Medium-Old) (n = 14)	*P* Value
Male, n (%)	83 (48.5%)	30 (45.5%)	16 (42.1%)	30 (56.6%)	7 (50.0%)	NS
Age, years (range)	52 ± 19 (20–84)	31 ± 7 (20–44)	55 ± 6* (45–64)	70 ± 3*^#^ (65–74)	76 ± 1*^#§^ (75–84)	<0.001
Ethnicity, n (%)						0.001
Chinese	154 (90.1%)	53 (80.3%)	34 (89.5%)	53 (100%)	14 (100%)	
Malay	7 (4.1%)	5 (7.6%)	2 (5.3%)	0 (0%)	0 (0%)	
Indian	2 (1.2%)	1 (1.5%)	1 (2.6%)	0 (0%)	0 (0%)	
Others	8 (4.7%)	6 (9.1%)	1 (2.6%)	0 (0%)	0 (0%)	
Smoking, n (%)						0.001
Never	160 (93.6%)	65 (98.5%)	37 (97.4%)	46 (86.8%)	12 (85.7%)	
Current	8 (4.7%)	1 (1.5%)	1 (2.6%)	6 (11.3%)	0 (0%)	
Past	3 (1.8%)	0 (0%)	0 (0%)	1 (1.9%)	2 (14.3%)	
Height, cm	163 ± 9	165 ± 10	162 ± 7	162 ± 8	158 ± 7*	0.008
Weight, kg	61 ± 12	63 ± 15	64 ± 12	59 ± 9	55 ± 7	0.042
Body surface area, m^2^	1.66 ± 0.20	1.70 ± 0.25	1.69 ± 0.19	1.63 ± 0.15	1.54 ± 0.11*	0.022
Body mass index, kg/m^2^	23.0 ± 3.3	22.9 ± 3.5	24.0 ± 3.3	22.6 ± 2.9	22.2 ± 3.4	0.205
SBP, mmHg	135 ± 20	126 ± 15	130 ± 18	146 ± 20*^#^	146 ± 22*^#^	<0.001
DBP, mmHg	77 ± 10	75 ± 11	79 ± 9	79 ± 10	74 ± 8	0.056
Heart rate, bpm	76 ± 12	77 ± 12	74 ± 12	76 ± 13	79 ± 11	0.526
LV EDV index, ml/m^2^	69 ± 11	74 ± 10	70 ± 10	66 ± 10*	55 ± 8*^#§^	<0.001
LV ESV index, ml/m^2^	25 ± 7	28 ± 7	24 ± 5*	24 ± 7*	18 ± 4*^#§^	<0.001
LV stroke volume index, ml/m^2^	44 ± 7	45 ± 6	46 ± 8	42 ± 6	37 ± 7*^#§^	<0.001
LV ejection fraction, %	64 ± 6	62 ± 6	64 ± 6	64 ± 7	67 ± 6*	0.011
LV mass index, g/m^2^	45 ± 11	45 ± 11	47 ± 10	45 ± 11	39 ± 7	0.151
LV mass-to-volume ratio, g/ml	0.65 ± 0.16	0.61 ± 0.15	0.66 ± 0.15	0.69 ± 0.16*	0.72 ± 0.11	0.017
ED curvedness, mm^−1^	0.041 ± 0.004	0.040 ± 0.004	0.041 ± 0.004	0.040 ± 0.004	0.043 ± 0.003	0.072
ES curvedness, mm^−1^	0.068 ± 0.011	0.066 ± 0.011	0.069 ± 0.009	0.068 ± 0.011^§^	0.078 ± 0.012*^#§^	0.002
ED wall thickness, mm	4.92 ± 0.77	4.80 ± 0.78	4.92 ± 0.67	5.03 ± 0.80	5.01 ± 0.80	0.425
ES wall thickness, mm	7.75 ± 1.22	7.46 ± 1.20	7.88 ± 0.92	7.95 ± 1.34	8.08 ± 1.42	0.089
ED wall stress index	2.72 ± 0.46	2.83 ± 0.45	2.69 ± 0.44	2.69 ± 0.46	2.48 ± 0.46	0.049
ES wall stress index	0.99 ± 0.24	1.07 ± 0.23	0.93 ± 0.16*	0.96 ± 0.25*	0.83 ± 0.25*	<0.001
Peak systolic wall stress, 1000 N/m^2^	15.7 ± 3.6	16.0 ± 3.5	14.1 ± 2.0	16.6 ± 3.9^#^	14.2 ± 3.8	0.005
Area strain, %	69 ± 11	66 ± 10	71 ± 8	69 ± 12	79 ± 13*^#§^	<0.001

Data were represented as mean ± SD or percentage. SBP: systolic blood pressure; DBP: diastolic blood pressure; LV: left ventricle; ED: end-diastolic; ES: end-systolic; EDV: end-diastolic volume; ESV: end-systolic volume. *P* value is from one-way analysis of variance (ANOVA) across the four age groups with Bonferroni test, a *P* value of 0.05 was considered significant. *Significant difference compared to Young group; ^#^significant difference compared to Mid-age group; ^§^significant difference compared to Young-Old group.

**Table 2:** Comparison of demographics and 3D regional parameters by gender.

Parameters	Male (n = 83)	Female (n = 88)	*P* Value
Age, years	53 ± 19	50 ± 19	0.297
Height, cm	169 ± 7	157 ± 6	<0.001
Weight, kg	67 ± 12	56 ± 10	<0.001
Body surface area, m^2^	1.77 ± 0.19	1.55 ± 0.15	<0.001
Body mass index, kg/m^2^	23.5 ± 3.2	22.4 ± 3.2	0.026
Systolic blood pressure, mmHg	140 ± 18	130 ± 21	0.001
Diastolic blood pressure, mmHg	81 ± 9	73 ± 10	<0.001
Heart rate, bpm	77 ± 12	76 ± 12	0.423
LV end-diastolic volume index, ml/m^2^	71 ± 12	67 ± 10	0.013
LV end-systolic volume index, ml/m^2^	26 ± 7	24 ± 7	0.031
LV stroke volume index, ml/m^2^	45 ± 7	43 ± 6	0.058
LV ejection fraction, %	63 ± 6	64 ± 6	0.414
LV mass index, g/m^2^	51 ± 10	39 ± 7	<0.001
LV mass-to-volume ratio, g/ml	0.72 ± 0.16	0.59 ± 0.12	<0.001
ED curvedness (*C*_*ED*_), mm^−1^	0.039 ± 0.004	0.042 ± 0.004	<0.001
ES curvedness (*C*_*ES*_), mm^−1^	0.066 ± 0.011	0.071 ± 0.010	0.003
ED wall thickness (*WT*_*ED*_), mm	5.33 ± 0.61	4.53 ± 0.70	<0.001
ES wall thickness (*WT*_*ES*_), mm	8.20 ± 1.07	7.33 ± 1.21	<0.001
ED wall stress index (*σ*_*i,ED*_)	2.55 ± 0.38	2.89 ± 0.47	<0.001
ES wall stress index (*σ*_*i,ES*_)	0.96 ± 0.23	1.01 ± 0.24	0.114
Peak systolic wall stress (*σ*_*ES*_), 1000 N /m^2^	15.8 ± 3.7	15.6 ± 3.5	0.670
Area strain (*AS*), %	67 ± 11	72 ± 11	0.001

Data were represented as mean ± SD. LV: left ventricle; ED: end-diastolic; ES: end-systolic. *P* value is from two-sample independent T-test.

### LV geometry reconstruction and regional parameters calculation

**Figure 1:**
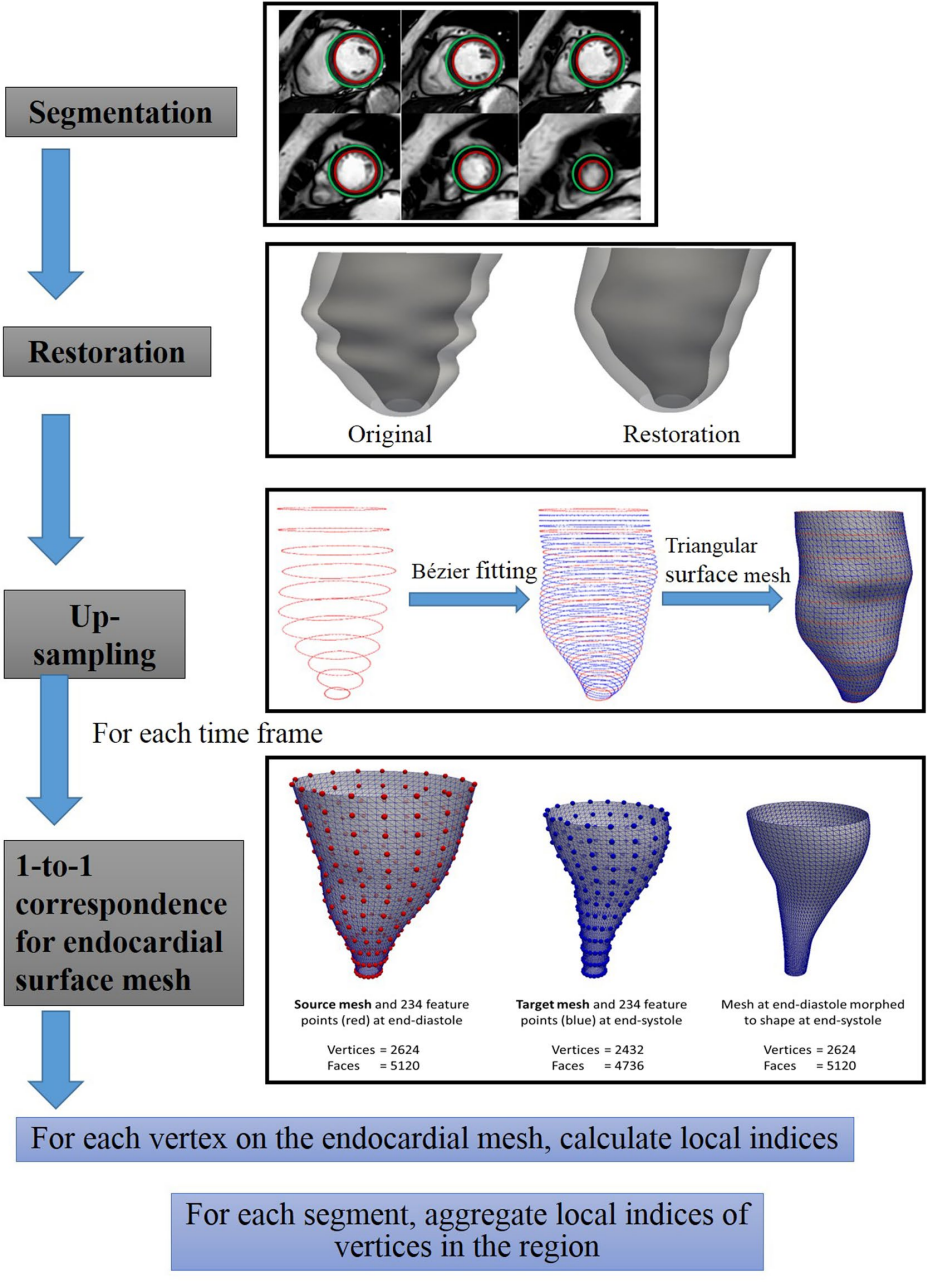
Workflow of the reconstruction process.

Segmented LV endocardial and epicardial contours of the stack of LV short-axis images from QMass (Medis, Leiden, the Netherlands) were imported into our in-house software (CardioWerkz) to reconstruct the 3D LV geometry. Figure 1 illustrates the workflow for the reconstruction process and the ensuing computation of associated 3D regional parameters: curvedness at ED and ES (*C*_*ED*_ and *C*_*ES*_), wall thickness at ED and ES (*WT*_*ED*_ and *WT*_*ES*_), curvature-based wall stress index at ED and ES (*σ*_*i,ED*_ and *σ*_*i,ES*_), peak systolic wall stress (*σ*_*ES*_), and area strain (*AS*). Detailed computations of these parameters are given in Methods section.

### Comparison of 3D regional parameters by age and gender

**Figure 2:**
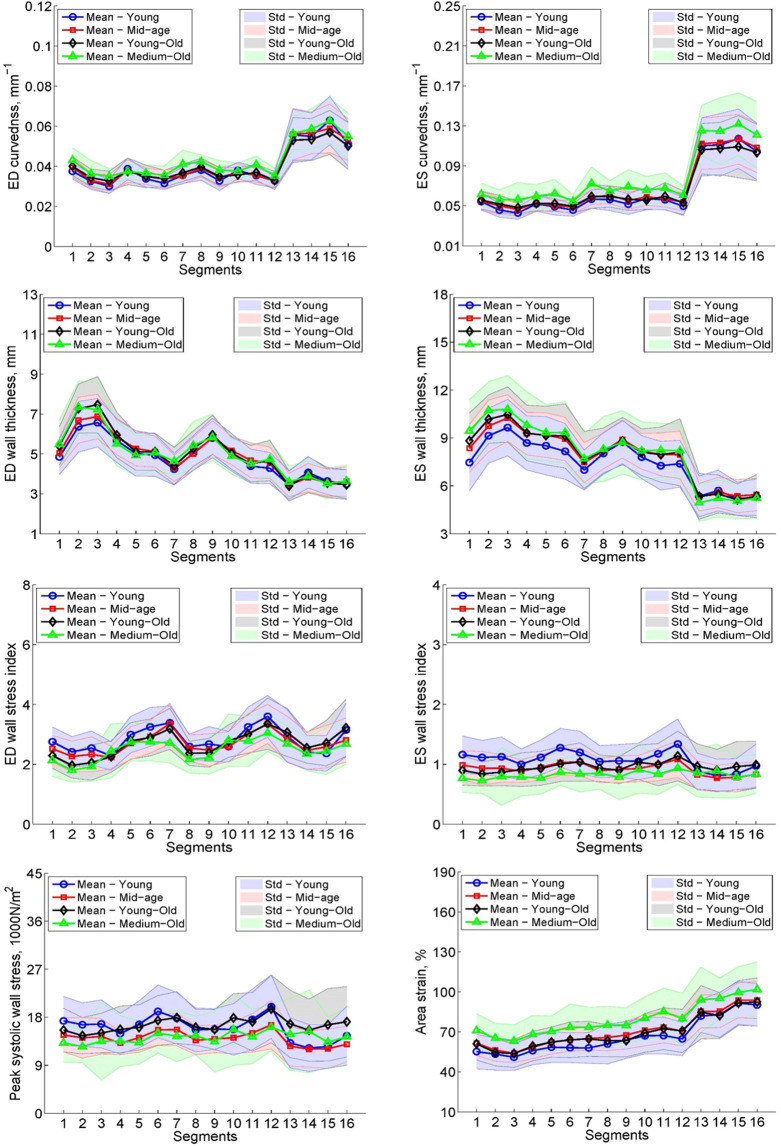
Shaded error plots for the four age groups of regional parameters vs segment. First row: curvedness at ED (left) and ES (right) vs segments, second row: wall thickness at ED (left) and ES (right) vs segments, third row: wall stress index at ED (left) and ES (right) vs segments, last row: peak systolic wall stress (left) and area strain (right) vs segments. Young: ≤44 years; Mid-age: 45–64 years; Young-Old: 65–74 years; Medium-Old: 75–84 years. ED: end-diastole; ES: end-systole; Std: standard deviation. Solid line represents mean value, shaded region represents standard deviation.

3D regional parameters for the four age groups are tabulated in Table 1. ANOVA indicated statistically significant differences among four age groups for *C*_*ES*_, *σ*_*i,ED*_, *σ*_*i,ES*_, *σ*_*ES*_ and *AS* (all *P* < 0.05). There were significantly higher *C*_*ES*_ and *AS* for those aged 75–84 years compared with the other three age groups (*P* < 0.05). The 45–64 years, 65–74 years and 75–84 years age groups showed significantly lower *σ*_*i,ES*_ compared with the ≤44 years age group. Shaded error plots (mean ± SD) for *C*_*ED*_, *C*_*ES*_, *WT*_*ED*_, *WT*_*ES*_, *σ*_*i,ED*_, *σ*_*i,ES*_, *σ*_*ES*_ and *AS* for individual LV wall segments among the age groups are given in Figure 2.

**Figure 3:**
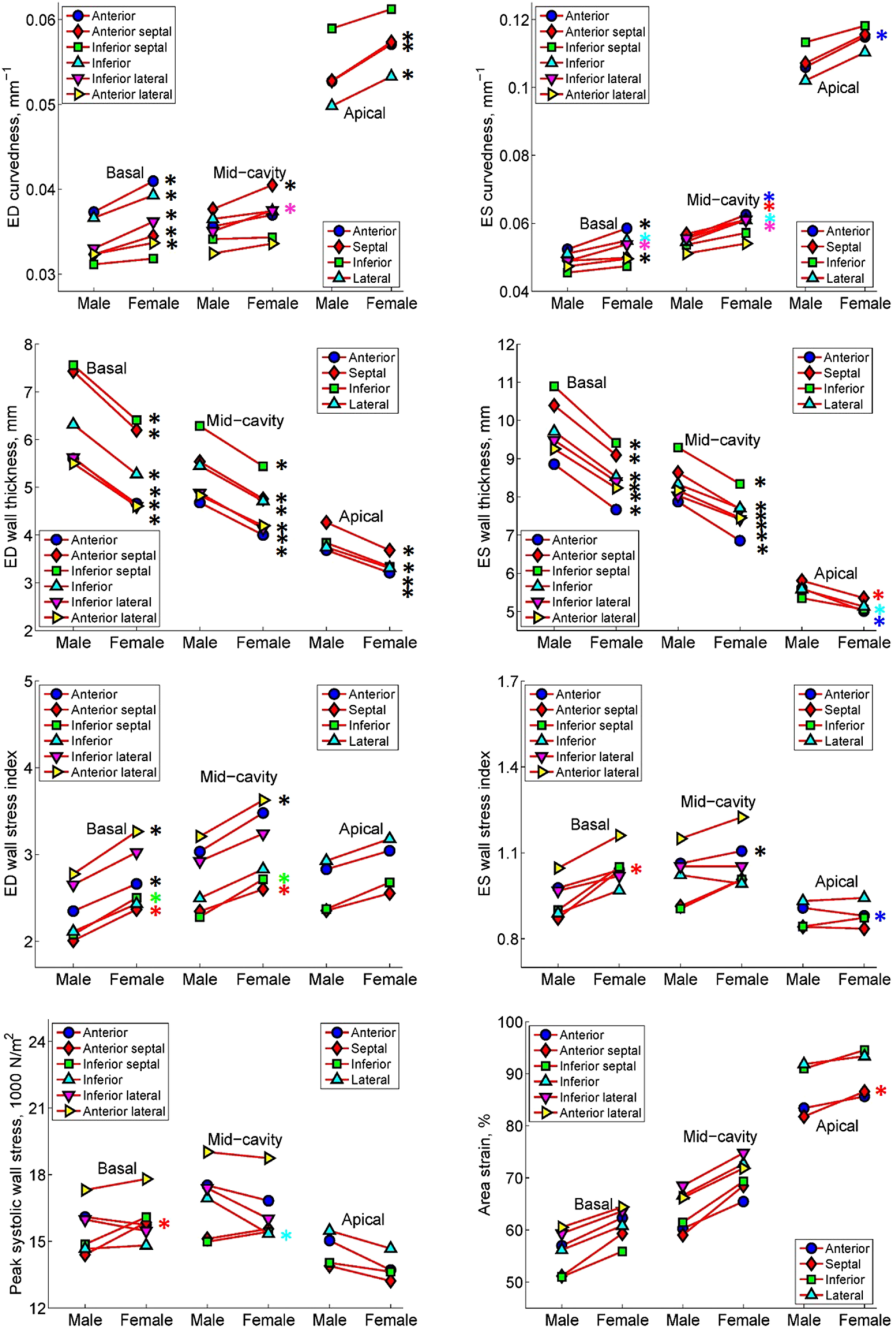
Regional segment comparison between male and female for regional parameters. Curvedness at ED and ES (first row), wall thickness at ED and ES (second row), wall stress index at ED and ES (third row), peak systolic wall stress (last row, left) and area strain (last row, right). ED: end-diastole; ES: end-systole. *denotes significant difference with *P* < 0.05 (colors of * correspond to segment colors if points overlap).

Comparisons of 3D regional parameters between males and females are presented in Table 2. *C*_*ED*_, *C*_*ES*_, *σ*_*i,ED*_ and *AS* were significantly larger in females (*C*_*ED*_, 0.042 ± 0.004 mm^−1^; *C*_*ES*_, 0.071 ± 0.010 mm^−1^; *σ*_*i,ED*_, 2.89 ± 0.47; *AS*, 72 ± 11%) compared with males (*C*_*ED*_, 0.039 ± 0.004 mm^−1^; *C*_*ES*_, 0.066 ± 0.011 mm^−1^; *σ*_*i,ED*_, 2.55 ± 0.38; *AS*, 67 ± 11%) (all *P* ≤ 0.003). No significant differences were seen for *σ*_*i,ES*_ and *σ*_*ES*_ between genders (male vs female: *σ*_*i,ES*_, 0.96 ± 0.23 vs 1.01 ± 0.24; *σ*_*ES*_, 15.8 ± 3.7 vs 15.6 ± 3.5 1000 N/m^2^). Gender comparisons of *C*_*ED*_, *C*_*ES*_, *WT*_*ED*_, *WT*_*ES*_, *σ*_*i,ED*_, *σ*_*i,ES*_, *σ*_*ES*_ and *AS* in individual LV segments are presented in Figure 3. Per segment comparisons of *C*_*ED*_ and *C*_*ES*_, showed significant differences at the following segments: basal anterior (Segment 1); inferior, inferior lateral and anterior lateral (Segments 4–6); mid anterior septal (Segment 8); inferior lateral (Segment 11); and apical anterior (Segment 13) (all *P* < 0.05). For wall thickness, females exhibited significantly thinner myocardial walls than males at both ED and ES for all segments (all *P* < 0.05) except for apical inferior at ES phase. For *σ*_*ES*_, with the exception of basal anterior septal (Segment 2) and mid inferior (Segment 10), no differences were found at other segments or aggregated values at basal, mid-cavity and apical levels. The basal and mid-cavity anterior lateral segments, and the apical lateral segment, had the largest *σ*_*ES*_. There were no differences between males and females in *AS*, with the exception of the apical septal segment (Segment 14).

### Interaction of age × gender with 3D regional parameters

**Figure 4:**
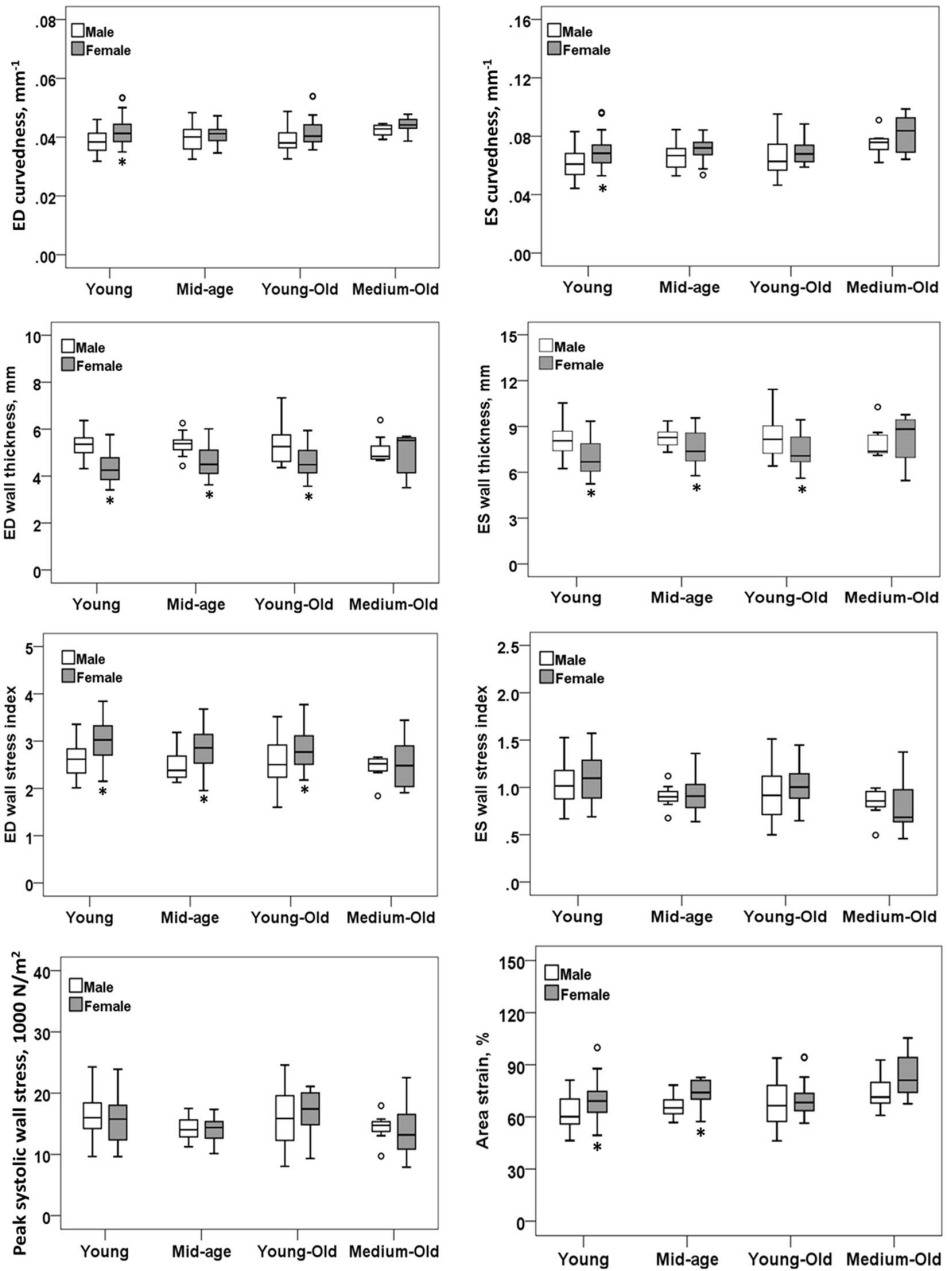
Box plots between male and female separated by four age groups. First row: curvedness at ED (left) and ES (right), second row: wall thickness at ED (left) and ES (right), third row: wall stress index at ED (left) and ES (right), last row: peak systolic wall stress (left) and area strain (right). Young: ≤44 years; Mid-age: 45–64 years; Young-Old: 65–74 years; Medium-Old: 75–84 years. ED: end-diastole; ES: end-systole. The outliers are plotted individually using ‘o’ symbol, *denotes significant difference with *P* < 0.05.

**Figure 5:**
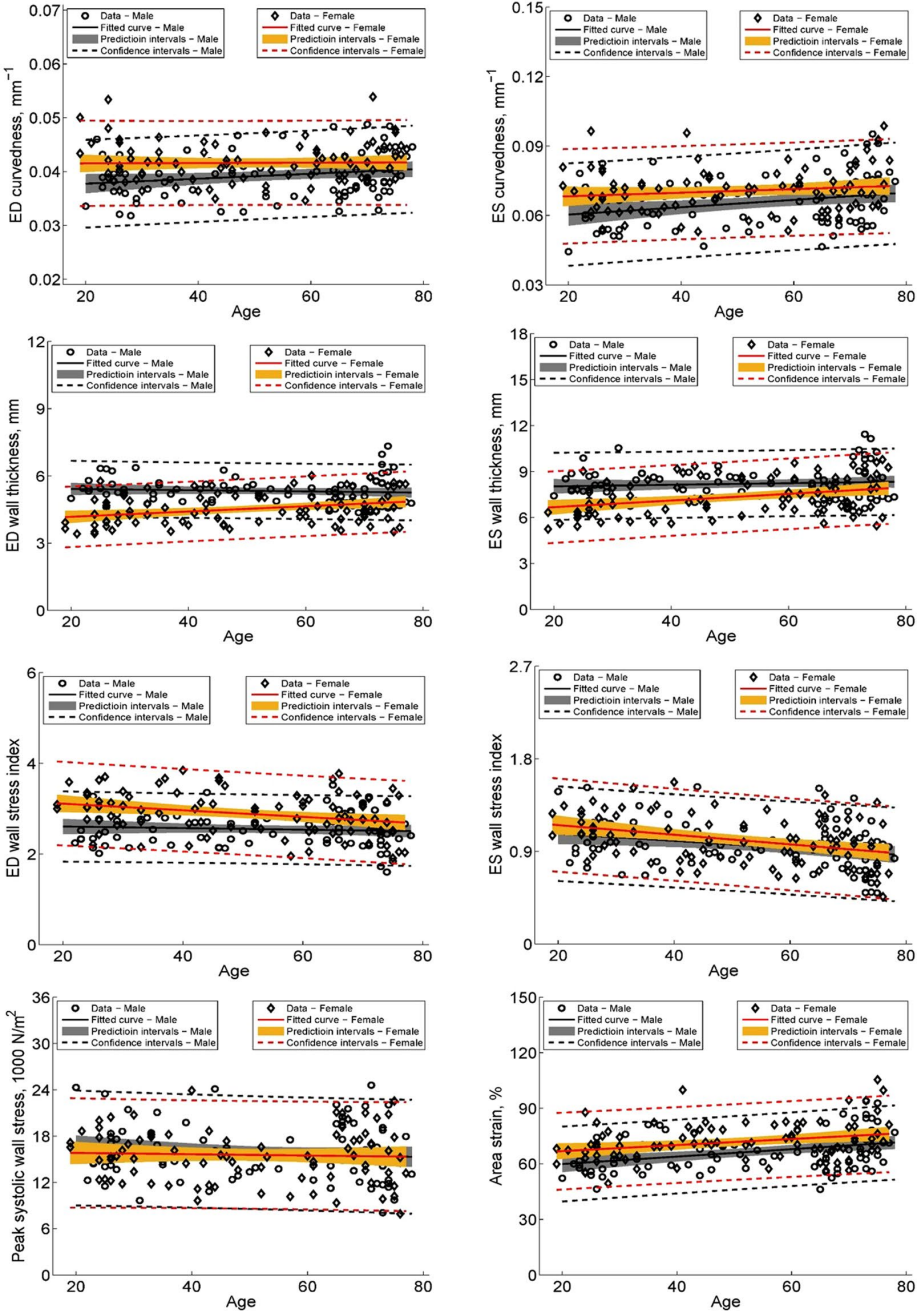
Linear regression plots of regional parameters vs age. First row: curvedness at ED (left) and ES (right) vs age, second row: wall thickness at ED (left) and ES (right) vs age, third row: wall stress index at ED (left) and ES (right) vs age, last row: peak systolic wall stress (left) and area strain (right) vs age. ED: end-diastole; ES: end-systole. Dash line represents 95% confidence interval (CI), shaded region represents 95% CIs for predicted values with linear regression.

Table 3 summarizes the results of 3D regional parameters stratified by gender and age groups (≤44, 45–64, 65–74 and 75–84 years). *C*_*ED*_ (*P* = 0.0067), *C*_*ES*_ (*P* = 0.0001) and *AS* (*P* < 0.0001) increased significantly with age, while *σ*_*i,ED*_ (*P* = 0.0160) and *σ*_*i,ES*_ (*P* = 0.0011) decreased with age. Results of male vs female comparisons of ANOVA least squares means are as follows: *C*_*ED*_ (0.040 vs 0.042 mm^−1^, *P* = 0.0042), *C*_*ES*_ (0.068 vs 0.073 mm^−1^, *P* = 0.0077), *σ*_*i,ED*_ (2.52 vs 2.81, *P* = 0.0002) and *AS* (68 vs 74%, *P* = 0.0006) were significantly lower in males than females; conversely, *WT*_*ED*_ (5.29 vs 4.62 mm, *P* < 0.0001) and *WT*_*ES*_ (8.17 vs 7.53 mm, *P* = 0.0022) were significantly higher in males than females. No gender differences were found in least squares means (male vs female) for *σ*_*i,ES*_ (0.93 vs 0.97, *P* = 0.344) or *σ*_*ES*_ (15.3 vs 15.2 1000 N/m^2^, *P* = 0.808). Figure 4 presents box plots of *C*_*ED*_, *C*_*ES*_, *WT*_*ED*_, *WT*_*ES*_, *σ*_*i,ED*_, *σ*_*i,ES*_, *σ*_*ES*_ and *AS* vs gender stratified by age group. Only in the ≤44 years group did females have significantly higher *C*_*ED*_ and *C*_*ES*_ than males. Except for the 75–84 years group, females had significantly smaller *WT*_*ED*_ and *WT*_*ES*_ and larger *σ*_*i,ED*_ than males in the other three age groups. Increased *AS* was observed in females for the ≤44 and 45–64 years groups (*P* < 0.05). Age × gender interactions were non-significant for all parameters, indicating relative constancy in differences between males and females across age categories. No significant differences were detected among age categories with respect to *WT*_*ED*_ (*P* = 0.626) and *WT*_*ES*_ (*P* = 0.133), indicating no significant effect of age on wall thickness. Significant differences in *σ*_*ES*_ were observed among the four age groups (*P* = 0.0051), while associations with age (*P* = 0.348) and gender differences (*P* = 0.808) were non-significant. Plotted in Figure 5 are linear regressions of *C*_*ED*_, *C*_*ES*_, *WT*_*ED*_, *WT*_*ES*_, *σ*_*i,ED*_, *σ*_*i,ES*_, *σ*_*ES*_ and *AS* vs age stratified by gender showing 95% confidence intervals and prediction intervals.

**Table 3:** Summary of results from statistical analysis on 3D regional parameters by age category, mean (SD).

Parameters	Gender	Age category (years)				LS Mean, Gender	*P* Value
		≤44 (Young) (n = 66, M/F 30/36)	45–64 (Mid-age) (n = 38, M/F 16/22)	65–74 (Young-Old) (n = 53, M/F 30/23)	75–84 (Medium-Old) (n = 14, M/F 7/7)		ANOVA main effect: Age Gender Interaction (Age × Gender) Linear trend: Age
ED curvedness (*C*_*ED*_), mm^−1^	M	0.038 (0.004)	0.040 (0.005)	0.039 (0.004)	0.042 (0.002)	0.04	A: 0.0460
							G: 0.0042
	F	0.042 (0.004)*	0.041 (0.003)	0.042 (0.004)	00044 (0.003)	0.042	I: 0.4315
	Pooled ^1^	0.04	0.041	0.04	0.043		LT: 0.0067
							Slope = 0.00002 (*P* = 0.2464)
ES curvedness (*C*_*ES*_), mm^−1^	M	0.062 (0.010)	0.066 (0.009)	0.066 (0.013)	0.075 (0.009)	0.068	A: 0.0011
							G: 0.0077
	F	0.070 (0.010)*	0.070 (0.008)	0.069 (0.009)	0.081 (0.014)	0.073	I: 0.7227
	Pooled	0.066	0.068	0.068	0.078		LT: 0.0001
							Slope = 0.00011 (*P* = 0.0160)
ED wall thickness (*WT*_*ED*_), mm	M	5.37 (0.50)	5.34 (0.43)	5.32 (0.79)	5.13 (0.65)	5.29	A: 0.6258
							G: < 0.0001
	F	4.34 (0.66)*	4.62 (0.66)*	4.65 (0.66)*	4.90 (0.97)	4.62	I: 0.1605
	Pooled	4.85	4.98	4.99	5.01		LT: 0.4127
							Slope = 0.00617 (*P* = 0.0484)
ES wall thickness (*WT*_*ES*_), mm	M	8.09 (0.92)	8.24 (0.54)	8.34 (1.40)	8.02 (1.14)	8.17	A: 0.1325
							G: 0.0022
	F	6.94 (1.16)*	7.62 (1.06)*	7.43 (1.10)*	8.13 (1.75)	7.53	I: 0.2596
	Pooled	7.51	7.93	7.89	8.07		LT: 0.1110
							Slope = 0.01517 (*P* = 0.0022)
ED wall stress index (*σ*_*i,ED*_)	M	2.61 (0.36)	2.47 (0.30)	2.55 (0.45)	2.43 (0.29)	2.52	A: 0.0479
							G: 0.0002
	F	3.01 (0.44)*	2.85 (0.47)*	2.85 (0.44)*	2.53 (0.62)	2.81	I: 0.6604
	Pooled	2.81	2.66	2.7	2.48		LT: 0.0160
							Slope = −0.00521 (*P* = 0.0052)
ES wall stress index (*σ*_*i,ES*_)	M	1.04 (0.24)	0.90 (0.10)	0.93 (0.27)	0.84 (0.17)	0.93	A: 0.0004
							G: 0.3442
	F	1.10 (0.23)	0.94 (0.20)	1.00 (0.21)	0.82 (0.33)	0.97	I: 0.9206
	Pooled	1.07	0.92	0.96	0.83		LT: 0.0011
							Slope = −0.00417 (*P* < 0.0001)
Peak systolic wall stress (*σ*_*ES*_), 1000 N/m^2^	M	16.4 (3.8)	14.2 (1.8)	16.3 (4.1)	14.4 (2.5)	15.3	A: 0.0051
							G: 0.8076
	F	15.7 (3.3)	13.9 (2.2)	16.9 (3.5)	14.1 (5.0)	15.2	I: 0.7890
	Pooled	16.1	14.1	16.6	14.2		LT: 0.3482
							Slope = −0.01343 (*P* = 0.3565)
Area strain (*AS*), %	M	63 (9)	66 (6)	69 (12)	74 (11)	68	A: 0.0002
							G: 0.0006
	F	69 (10)*	74 (7)*	70 (10)	84 (14)	74	I: 0.3250
	Pooled	66	67	67	74		LT: < 0.0001
							Slope = 0.00169 (*P* = 0.0001)

SD: standard deviation; ANOVA: analysis of variance; ED: end-diastolic; ES: end-systolic; LS: least squares; LT: linear trend. *significant difference between male and female, bolded text indicates a significant *P* value.

### Associations of age and gender with 3D regional parameters

We also investigated associations of age and gender vs LV function and 3D regional parameters, and the correlation coefficients are given in Table 4. Significant negative association with age was observed for both males and females for LV EDV index and LV ESV index, while LV EF was positively associated with age (*P* < 0.05 for both genders). However, only females had significant positive association with age for LV mass-to-volume ratio (*r* = 0.453, *P* < 0.01). Correlation of *C*_*ES*_ with age was higher for males than females (*r* = 0.271, *P* < 0.05 vs *r* = 0.142, *P* = NS), while negative association of *σ*_*i,ES*_ with age was more pronounced in females than in males (*r* = −0.363 vs *r* = −0.285, both *P* < 0.01). Correlation between wall thickness and age was higher in females vs. males at both ED (*r* = 0.313, *P* < 0.01 vs *r* = −0.085, *P* = NS) and ES (*r* = 0.331, *P* < 0.01 vs *r* = 0.092, *P* = NS). No significant correlation was observed between *σ*_*ES*_ and age in either males or females (*r* = −0.104 vs *r* = −0.047, both *P* = NS). For *AS*, males were more correlated with age than females (*r* = 0.362 vs *r* = 0.284, both *P* < 0.01).

**Table 4:** Correlation of age with left ventricular (LV) function measurements and 3D regional parameters.

Parameters	All subjects (n = 171)	Male (n = 83)	Female (n = 88)
LV end-diastolic (ED) volume index, ml/m^2^	−0.408**	−0.438**	−0.433**
LV end-systolic (ES) volume index, ml/m^2^	−0.404**	−0.413**	−0.443**
LV ejection fraction, %	0.255**	0.232*	0.291**
LV mass index, g/m^2^	−0.013	−0.234*	0.147
LV mass-to-volume ratio, g/ml	0.267**	0.108	0.453**
ED curvedness (*C*_*ED*_), mm^−1^	0.089	0.217*	0.014
ES curvedness (*C*_*ES*_), mm^−1^	0.184*	0.271*	0.142
ED wall thickness (*WT*_*ED*_), mm	0.151*	−0.085	0.313**
ES wall thickness (*WT*_*ES*_), mm	0.233**	0.092	0.331**
ED wall stress index (*σ*_*i,ED*_)	−0.213**	−0.088	−0.286**
ES wall stress index (*σ*_*i,ES*_)	−0.331**	−0.285**	−0.363**
Peak systolic wall stress (*σ*_*ES*_), 1000 N/m^2^	−0.073	−0.104	−0.047
Area strain (*AS*), %	0.293**	0.362**	0.284**

*Correlation significant at the 0.05 level; **correlation significant at the 0.01 level.

### Intra- and inter- observer agreement

Table 5 shows the intra- and inter-observer variability for *C*_*ED*_, *C*_*ES*_, *WT*_*ED*_, *WT*_*ES*_, *σ*_*i,ED*_, *σ*_*i,ES*_, *σ*_*ES*_ and *AS*, respectively. Intra-observer intraclass correlation coefficient (ICC) and coefficient of variation (CV) were 0.949–0.993 and 3.3–8.8%, and inter-observer ICC and CV were 0.924–0.991 and 3.9–10.1%. Both intra- and inter-observer variability have small biases and limits of agreement.

**Table 5:** Intra- and inter-observer reproducibility of 3D regional parameters.

Parameters	Intra-observer (n = 20)			Inter-observer (n = 20)		
	ICC	Bias (limits of agreement)	CV, %	ICC	Bias (limits of agreement)	CV, %
ED curvedness (*C*_*ED*_), mm^−1^	0.993	0.0007 (−0.0027, 0.0040)	3.3	0.991	0.0011 (−0.0026, 0.0047)	3.9
ES curvedness (*C*_*ES*_), mm^−1^	0.988	−0.0020 (−0.0101, 0.0142)	6.7	0.985	−0.0021 (−0.0112, 0.0157)	7.5
ED wall thickness (*WT*_*ED*_), mm	0.981	0.02 (−0.74, 0.78)	4.9	0.976	0.16 (−0.70, 1.01)	5.9
ES wall thickness (*WT*_*ES*_), mm	0.985	0.01 (−1.10, 1.11)	4.5	0.969	0.06 (−1.50, 1.62)	6.3
ED wall stress index (*σ*_*i,ED*_)	0.952	−0.03 (−0.49, 0.43)	7.0	0.955	−0.12 (−0.57, 0.33)	7.6
ES wall stress index (*σ*_*i,ES*_)	0.949	0.02 (−0.74, 0.78)	8.5	0.924	−0.02 (−0.24, 0.21)	10.1
Peak systolic wall stress (*σ*_*ES*_), 1000 N/m^2^	0.951	−0.24 (−3.53, 3.05)	8.8	0.931	−0.27 (−4.02, 3.49)	10.0
Area strain (*AS*), %	0.989	0.01 (−0.06, 0.09)	3.8	0.980	0.01 (−0.09, 0.11)	5.0

ED: end-diastole; ES: end-systole; ICC: intraclass correlation coefficient; CV: coefficient of variation.

## Discussion

Our study establishes the age- and gender-specific CMR reference ranges for 3D regional LV curvedness, thickness, wall stress index and area strain that can be automatically calculated from the reconstructed LV geometry. Age was positively associated with curvedness and area strain more in males, while negatively correlated with wall stress index more in females. On average, females exhibited significantly greater curvedness, wall stress index and area strain, but reduced wall thickness compared to males. Peak systolic wall stress showed no gender differences across the entire cohort or among age groups, and was not associated with age. Importantly, age × gender interaction effects were non-significant for all parameters.

Regional parameters derived from the reconstructed LV geometry can provide new insights into the local mechanics that are not apparent from the 2D approach. Transthoracic echocardiography (TTE) is the standard and commonest clinical imaging modality for measuring LV wall thickness at the ED phase from the left parasternal long-axis view, but it is highly dependent on imaging plane obliquity, acoustic window and operator^10^. In Hindieh, *et al*.^10^, discordant measurements between TTE and CMR in maximal LV wall thickness at the parasternal long- and short-axis views were present in a significant subset of HCM patients because of TTE technique limitations. CMR has been shown to be superior to TTE in measuring LV chamber dimensions and wall thickness owing to image quality and accuracy with better reproducibility than TTE^11^. CMR 2D normal values for ED wall thickness were established from 300 participants (ages 45–94) free of cardiovascular disease in the Multi-Ethic Study of Atherosclerosis (MESA) cohort, and measurements were performed on both cine short- and long-axis images using QMass V.7.2 (Medis Medical Imaging Systems, Netherlands)^12^. Compared with their results, our wall thickness values tended to be smaller. The discrepancy could be explained by: (1) LV wall thickness increases with aging, and participants in their paper were middle-aged or older compared with our relatively younger cohort (mean age 66 ±  9 years vs 52  ±  19 years, respectively); (2) participants in the MESA study had larger LV mass index (and thereby thicker LV myocardial wall) on average compared with our Asian cohort (85.1 ± 15.2 vs 51 ± 10 g/m^2^ in males and 66.9 ± 10.9 vs 39 ± 7 g/m^2^ in females)^13^; and 3) variations in measurement methodology. In our study, wall thickness was computed from a 3D reconstructed LV model and is the mean of the wall thicknesses of all points within the segment. The wall thickness is defined as the length of a ray with origin at a point of interest on the endocardial surface that is directed towards, and terminates at its intersection with the epicardial surface, and is neither dependent on the location of a center point nor imaging frame of reference^2^. This ray is not in the same plane as, and is always shorter than the distance measured on, standard 2D short- and long-axis slices. It approximates the “true” wall thickness in 3D space, and unlike 2D measurements, is independent of inaccuracies in slice positioning. Therefore, as suggested by Kawel, *et al*.^12^, it is critical to report the measurement technique along with the measured parameters. In addition, from the 16 segment plots in Figure 3, larger values of wall thickness are seen in segments containing the interventricular septum (IVS) in both males and females, indicating an asymmetry of LV myocardium that is similarly seen on 2D measurements^12^.

2D curvature has been proposed for characterizing LV local shapes in different HCM subtypes using independent coordinates method^14^. In pulmonary hypertension patients, the ratio of IVS curvature and free wall curvature at the ES phase by the three-point arc method was used to predict right ventricular (RV) systolic pressure^15^. Several shape descriptors have been proposed to characterize and quantify the differential properties of surfaces. The most common ones —first mentioned by Koenderink and Van Doorn^16^— are Gaussian curvature (*K* = *k*_1_*k*_2_), mean curvature [*H* = (*k*_1_ + *k*_2_)/2] and curvedness $(C=\sqrt{{k}_{1}^{2}+{k}_{2}^{2}}/2)$. Mean curvature *H* measured on 3D echocardiography (3DE) has been proposed by Addetia, *et al*.^17^ to discriminate normal pressure from right ventricular pressure overload in pulmonary arterial hypertension patients, and normal 3DE values of RV regional curvature indices were established by the same group^18^. In a retrospective study of 416 inpatients using 2DE and 3DE, regional curvature *H* provided additional mortality risk prediction beyond global longitudinal strain and LV EF^19^. A serious limitation of curvature analysis is that *H* will be zero at the saddle point even when the surface is curved. Similarly, Gaussian curvature *K* is zero at the parabolic line on a toroidal surface even as the surface is actually curved^2^. In contrast, point curvedness is zero only when the surface is flat, and varies monotonically with the magnitude of surface curve characteristics. In Maffessanti, *et al*.^20^, curvedness, normalized to instantaneous LV size, was calculated using prototype software (4D-LV Analysis MR, TomTec Imaging Systems, Unterschleissheim, Germany) and compared between segments for subjects with normal LV function (n = 14), dilated cardiomyopathy (n = 15) and ischemic heart failure (n = 15) using CMR images. The study size does not permit the determination of normal ranges of curvedness parameters. In contrast, our study, which recruited equal proportions of healthy male and female subjects stratified by age, is sizeable and able to establish gender- and age-specific normal ranges. Our results show that curvedness at ES is positively associated with age, indicating that the endocardial surface at the ES phase becomes more spherical during the ageing process.

LV wall stress is proportional to wall radius and inversely proportionate to wall thickness according to Laplace’s law^21^. Numerous formulas have been proposed to evaluate wall stress that assume the LV as an ideal spherical, spheroidal or ellipsoidal shape^22–25^. Some of these geometrical limitations can be overcome by finite element analysis (FEA) which is a numerical and engineering technique for solving complicated structural problems. The characteristics of wall stress have been applied in translational research to analyze LV mechanics^26,27^. From the curvature-based wall stress index formula, increases in curvedness and wall thickness with ageing (decrease of radius-to-thickness ratio *R*/*WT*) will contribute to decreases in wall stress index, which is what we show in Table 4 (*r* = −0.213 for *σ*_*i,ED*_ and *r* = −0.331 for *σ*_*i,ES*_). However, for peak systolic wall stress *σ*_*ES*_, which incorporates systolic blood pressure into the ES wall stress index, no association with age was found. Across each age group, *σ*_*ES*_ first decreased in the 45–64 years age group due to the decrease in radius-to-thickness ratio, then increased in the 65–74 years group because *R*/*WT* is counterbalancing the somewhat elevated systolic blood pressure. Even though there was no clinical history of hypertension in the study population, *σ*_*ES*_ eventually dropped in the 75–84 years group. However, *σ*_*ES*_ exhibited no significant differences between males and females for all subjects, which implies that *σ*_*ES*_ can be thought of as an intrinsic contractility index that is independent of gender that can potentially be helpful for longitudinal and/or therapeutic monitoring in disease applications.

Area strain *AS* reflects endocardial surface deformation during contraction and relaxation and integrates the regional changes from the circumferential, longitudinal and radial directions. Previous 3D echocardiography studies used percentage change in area from the original dimensions to quantify *AS*^28,29^. In contrast, our calculation was based on the natural log (ln) function of the ratio of surface area at ES and ED^4^. We found a significant gender difference in *AS* with greater deformation magnitude in females and greater rates of increase with age in males (*r* = 0.362). A similar trend was found for peak systolic longitudinal strain evaluated by CMR feature-tracking (CMR FT)^30^, an emerging technique for quantitating myocardial deformation in both clinical and research settings. Normal strain ranges using this approach have been presented^1,30–32^, however, only 2D basal, mid-cavity and apical LV short-axis slices were used to calculate global circumferential and radial strains, and only three standard LV long-axis views for global longitudinal strain. Hyperelastic warping is another technique employed to characterize cardiac motion and function measurement in finite deformation continuum mechanics and image-based data^33^, and this method has been applied to characterizing pulmonary hypertension^34–36^ and heart failure with preserved ejection fraction^37^. Strain measurements from this technique are based on the reconstruction of a 3D biventricular model that can extract circumferential, longitudinal and radial strains for the LV, RV and septum simultaneously.

**Figure 6:**
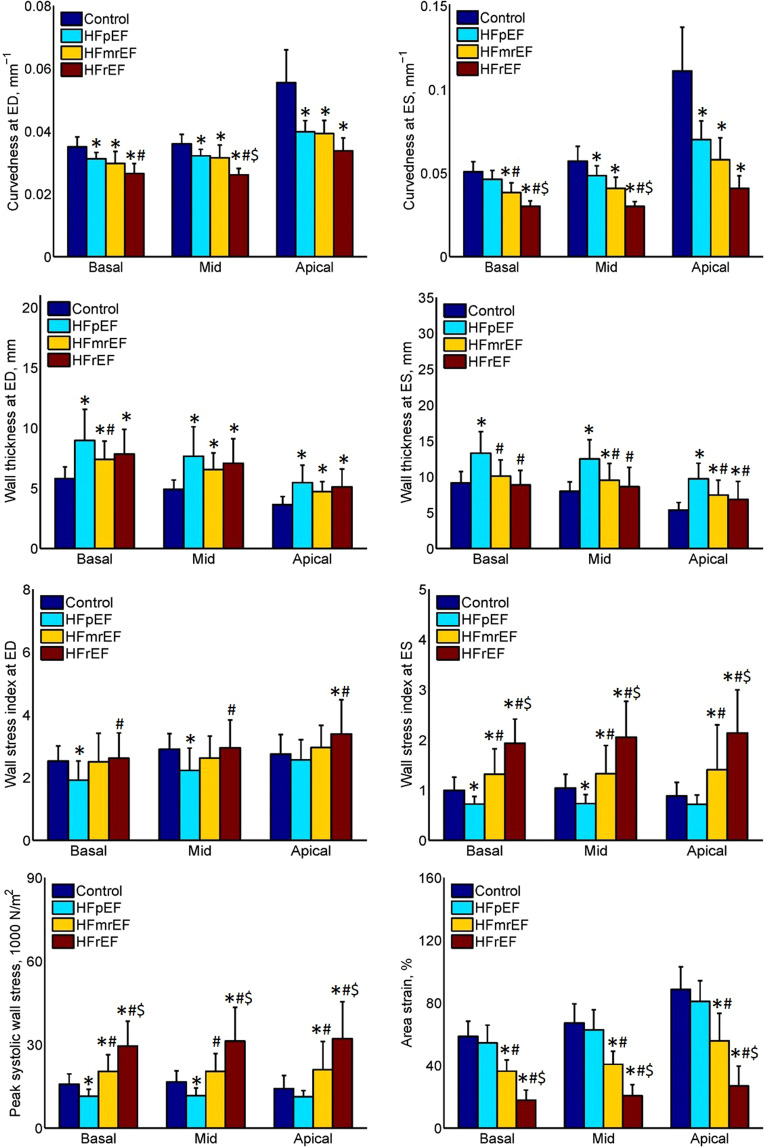
Bar chart comparison of aggregated means at basal, mid and apical levels between Control (n=171), HFpEF (n=10), HFmrEF (n=10) and HFrEF (n=10) groups. First row: curvedness at ED (left) and ES (right), second row: wall thickness at ED (left) and ES (right), third row: wall stress index at ED (left) and ES (right), last row: peak systolic wall stress (left) and area strain (right). HF: heart failure; EF: ejection fraction; HFpEF: HF with preserved EF; HFmrEF: HF with mid-range EF; HFrEF: HF with reduced EF; ED: end-diastole; ES: end-systole. *Significant difference compared to Control; ^#^significant difference compared to HFpEF; ^$^significant difference compared to HFmrEF.

To demonstrate the clinical utility of normal reference values for curvature-based LV parameters, three patient groups —HF with reduced EF (HFrEF, EF < 40%; n =10, 7 males, mean age 57 ± 11 years, range = 35–76 years); mid-range EF (HFmrEF, 40% ≤ EF < 50%; n =10, 7 males, mean age 59 ± 13 years, range = 38–75 years) and preserved EF (HFpEF, EF ≥ 50%; n =10, 8 males, mean age 58 ± 14 years, range = 37–77 years)— were evaluated using the 3D regional analysis described in this study. Comparisons of demographics and regional parameters in each group against the normal reference values are presented in Table 6. For LV volume indices, LVEF, LV mass index and all regional parameters, there were significant differences among Controls and the three HF patient groups (all *P* ≤ 0.008). Both ED and ES curvedness in the HF patient groups were smaller than in the normal references and decreased from HFpEF to HFmrEF to HFrEF, implying that the LV was becoming flatter with decrease in LV EF. LV myocardial wall was thicker in HF patient groups than normal group, and ES wall thickness increased from Control to HFrEF to HFmrEF to HFpEF. With the progressive reduction of EF, HF patients had a propensity for higher wall stress index at ES and peak systolic wall stress, with trend HFpEF to Control to HFmrEF to HFrEF. The HFrEF stress was about twice that in Controls and around 2.7 times that in HFpEF. This can be explained as follows: the normal or near normal chamber size and increased wall thickness relative to chamber dimension in HFpEF patients (concentric remodeling) resulted in reduced wall stress relative to Control. In contrast, in HFrEF, there is LV chamber dilatation (eccentric remodeling), with increase in LV radius^38^. However, the decrease of *AS* was from Control to HFpEF to HFmrEF to HFrEF. Moreover, HFmrEF and HFrEF were significantly different from Control, HFpEF and each other. The bar graphs in Figure 6 present aggregated values at the basal, mid and apical levels observed in Control (blue bars) and the three HF groups (HFpEF: cyan bars; HFmrEF: yellow bars; HFrEF: dark red bars) with curvedness (first row) at ED and ES (left and right, respectively), wall thickness (second row) at ED and ES, wall stress index (third row) at ED and ES, and peak systolic wall stress (last row, left) and area strain (last row, right). Special attention should be paid to the three wall stress parameters: in Control, the mid-level had the largest values whereas in the three HF patient groups, the largest values occurred at the apical level.

**Table 6:** Comparison of demographics and curvature-based left ventricular (LV) parameters between heathy subjects and heart failure patients.

Parameters	Control (n = 171)	HFpEF (n = 10)	HFmrEF (n = 10)	HFrEF (n = 10)	*P* Value
Age, years (range)	52 ± 19 (20–84)	58 ± 14 (37–77)	59 ± 13 (38–75)	57 ± 11 (35–76)	0.366
Male, n (%)	83 (48.5%)	8 (80.0%)	7 (70.0%)	7 (70.0%)	0.087
Height, cm	163 ± 9	163 ± 7	162 ± 7	165 ± 12	0.923
Weight, kg	61 ± 12	73 ± 14	73 ± 17*	82 ± 21*	<0.001
Body surface area, m^2^	1.66 ± 0.20	1.81 ± 0.20	1.80 ± 0.20	1.92 ± 0.29*	<0.001
Systolic blood pressure, mmHg	135 ± 20	135 ± 27	135 ± 22	130 ± 26	0.913
Diastolic blood pressure, mmHg	77 ± 10	73 ± 26	70 ± 13	82 ± 18	0.144
Heart rate, bpm	76 ± 12	65 ± 14*	74 ± 23	81 ± 16^#^	0.026
LV end-diastolic volume index, ml/m^2^	69 ± 11	84 ± 12*	97 ± 25*	125 ± 25*^#$^	<0.001
LV end-systolic volume index, ml/m^2^	25 ± 7	36 ± 6*	55 ± 14*^#^	92 ± 24*^#$^	<0.001
LV stroke volume index, ml/m^2^	44 ± 7	48 ± 7	42 ± 11	33 ± 6*^#$^	<0.001
LV ejection fraction, %	64 ± 6	56 ± 4*	43 ± 2*^#^	27 ± 7*^#$^	<0.001
LV mass index, g/m^2^	45 ± 11	71 ± 23*	68 ± 19*	75 ± 24*	<0.001
LV mass-to-volume ratio, g/ml	0.65 ± 0.16	0.85 ± 0.25*	0.72 ± 0.21	0.61 ± 0.16^#^	0.005
ED curvedness (*C*_*ED*_), mm^−1^	0.041 ± 0.004	0.034 ± 0.002*	0.033 ± 0.004*	0.028 ± 0.003*^#^	<0.001
ES curvedness (*C*_*ES*_), mm^−1^	0.068 ± 0.011	0.053 ± 0.007*	0.044 ± 0.007*	0.033 ± 0.004*^#^	<0.001
ED wall thickness (*WT*_*ED*_), mm	4.92 ± 0.77	7.59 ± 2.21*	6.39 ± 1.21*^#^	6.86 ± 1.88*	<0.001
ES wall thickness (*WT*_*ES*_), mm	7.75 ± 1.23	12.09 ± 2.61*	9.22 ± 2.12*^#^	8.27 ± 2.36^#^	<0.001
ED wall stress index (*σ*_*i,ED*_)	2.72 ± 0.46	2.19 ± 0.63*	2.66 ± 0.75	2.93 ± 0.88	0.008
ES wall stress index (*σ*_*i,ES*_)	0.99 ± 0.24	0.73 ± 0.15*	1.34 ± 0.60*^#^	2.03 ± 0.64*^#$^	<0.001
Peak systolic wall stress (*σ*_*ES*_), 1000 N/m^2^	15.7 ± 3.6	11.5 ± 2.3*	20.6 ± 6.6*^#^	30.8 ± 10.9*^#$^	<0.001
Area strain (*AS*), %	69 ± 11	64 ± 12	43 ± 9*^#^	21 ± 7*^#$^	<0.001

Data were represented as mean ± SD. HFpEF: heart failure with preserved ejection fraction; HFmrEF: heart failure with mid-range ejection fraction; HFrEF: heart failure with reduced ejection fraction; ED: end-diastole; ES: end-systole. *Significant difference compared to Control, ^#^significant difference compared to HFpEF, ^$^significant difference compared to HFmrEF.

These reference values were derived from a multi-ethnic Singaporean cohort with predominant Chinese ethnicity, which may limit the generalizability of the indices to Western populations. Our group has previously demonstrated that standard CMR parameters like chamber volumes and mass derived from a predominantly Chinese population were different than Western norms^9^. As such, it would not be surprising if reference ranges for curvedness-based parameters were to differ between ethnic groups. Fortunately, curvedness and the derived parameters can be determined expeditiously with post-processing of standard cine CMR images, and can therefore be applied retrospectively to extant research or clinical image repositories to generate references ranges in different populations and/or diverse disease states.

The sample size in the 75–84 years group was smaller than the other age groups owing to challenges in recruiting older subjects free of cardiovascular diseases, hypertension, diabetes and dyslipidemia. In the present study, manual delineation of endocardial and epicardial contours at ED and ES was needed to generate inputs for 3D geometric reconstruction, which is manually intensive. Incorporation of automatic segmentation^39–42^ and deep learning techniques^43–45^ may reduce processing time, is the next logic step. One of the limitations in our current reconstruction approach is the omission of the true apex (Segment 17 on the standard nomenclature) as it cannot be easily identified using the short-axis images due to thick (10 mm) slice acquisition. This may potentially lead to underestimation of LV volume. Another limitation concerns the tracking of LV chamber height, which shortens from ED to ES. LV chamber height is measured using the line joining the LV apex to the midpoint of the straight line connecting the two atrioventricular junctions, which assumes that the base of the LV is a flat plane orthogonal to the image defined solely by the two atrioventricular junction points. In reality, the LV basal surface is non-planar and the mitral valve is saddle-shaped. In our study, endocardial and epicardial borders at the base of the LV were manually contoured on short-axis cine CMR images, which is the routine in research and clinical laboratories. The left atrium (LA)-LV and LV-Aorta (AO) intersections are almost tangential to, and frequently not well-defined on, LV short-axis planes. A more optimal approach would be to reconstruct a whole heart model with the left atrium (LA) and aorta (AO) intact, and then to subsequently isolate the LV by truncating at the mitral annular and aortic planes. In a prior work, we acquired a series of 18 rotational slices at 10° angular equidistance in the LV long axis —the line extending from the LV apex to the center of mitral valve orifice— that depicts the mitral valve and annulus comprehensively^46^. Future work will be to develop an algorithm to construct a composite 3D LV+LA+AO geometric model using 18 rotational slices as the reference, and to compare volumetric measurements with the LV models reconstructed from routine CMR images.

One attractive feature of our CardioWerkz software is that it does not require additional image acquisition and only uses short- and long-axis CMR images acquired in routine clinical practice. The short-axis contours used for LV function measurements can be directly imported into CardioWerkz for LV regional parameter evaluation permitting retrospective study of various cohorts. The relatively complex structure of the RV has rendered it less studied compared with the LV. Normal values of RV regional curvature indices using 3D echocardiography have been established by Addetia, *et al*.^18^. However, acquisition of 3D RV echocardiographic data sets is quite challenging and often hampered by varying degrees of anterior wall dropout^17^. Using routine CMR to reconstruct 3D RV geometry and performing curvedness analysis has been reported in our prior study^7^. A future study will establish normal ranges of RV regional deformation using the same stacks of LV short-axis cine CMR images, which also cover the RV chamber, combined with automatic segmentation of RV endocardial contours^47,48^.

We have established normal values for 3D regional curvedness, wall thickness, wall stress index and *AS* for healthy subjects across a broad age spectrum, and investigated the associations with age and gender. Our approach is highly reproducible and automatic apart from contour extraction. The parameters in this study may shed light on the LV regional deformation with ageing.

## Methods

### Study population

Between July 2011 and July 2016, 444 healthy asymptomatic subjects (age range 20–87) without known cardiovascular disease were recruited from the community to undergo CMR. Those with prior history of hypertension, dyslipidemia, diabetes mellitus, and detected to have LV ejection fraction (EF) < 50% on CMR were excluded from analysis, leaving a final study sample of 171 subjects. The study was conducted in accordance with the Declaration of Helsinki and approved by the SingHealth Centralized Institutional Review Board. Written informed consent was obtained from all subjects.

As a clinical application of our normal ranges, we also included three heart failure (HF) patient groups —HF with reduced EF (HFrEF, EF < 40%; M/F: 7/3); mid-range EF (HFmrEF, 40% ≤ EF < 50%; M/F: 7/3) and preserved EF (HFpEF, EF ≥ 50%; M/F: 8/2). Inclusion criteria of HF required the presence of signs or symptoms of HF based on modified Framingham criteira^49^ and prior hospitalization with primary diagnosis of HF. Exclusion criteria for HF included specific subgroups of HF (e.g., amyloidosis, eosinophilic myocarditis, etc.) and isolated right heart disease.

### CMR acquisition and analysis

All subjects underwent CMR on a 3.0 Tesla system (Ingenia, Philips Healthcare, Best, the Netherlands) with a dStream Torso coil. Contiguous end-expiratory breath-held balanced fast field echo short-axis cine images covering the LV from base to apex were acquired along with routine 2-, 3- and 4-chamber long-axis cine images. Typical sequence parameters were: TR/TE 3/1 ms, flip angle 45°, slice thickness 10 mm for both short- and long-axis with 0.6 mm × 0.6 mm to 1.1 mm × 1.1 mm in-plane spatial resolution, pixel bandwidth 1797 Hz, field of view 280–450 mm, and frame rate was 30 frames per cardiac cycle.

Commercially available software QMass (Medis, Leiden, the Netherlands) was used for standard volumetric analysis. LV endocardial and epicardial contours were delineated manually at end-diastole (ED) and end-systole (ES) for each short-axis slice to determine LV end-diastolic volume and end-systolic volume, stroke volume (SV) and EF. Papillary and trabeculae muscles were included in the chamber volume calculation. LV mass was estimated at the ED phase as (epicardial volume − endocardial volume) × 1.05 g/ml. LV endocardial and epicardial contours in the remaining time phases were automatically tracked for each short-axis slice and manually corrected where needed.

### Reconstruction of 3D LV geometry

Segmented LV endocardial and epicardial contours of the stack of LV short-axis images from QMass were imported into our in-house software (CardioWerkz, version 0.9 beta) to reconstruct the 3D LV geometry. CardioWerkz is a proprietary in-house developed software comprising a suite of algorithms for geometrical reconstruction of the left heart and its subsequent analysis. The workflow for the reconstruction process and the ensuing computation of associated 3D regional parameters is illustrated in Figure 1. The key steps are: correction of any misalignment in the LV short-axis slices arising from patient motion using a shape-based energy minimization approach^50^; up-sampling of both endocardial and epicardial short-axis contours using a Bézier fitting algorithm to facilitate smooth surface reconstruction; and reconstruction of both LV endocardial and epicardial surfaces in the form of unstructured triangular meshes. Additionally, for LV endocardial surface meshes, we generated 1-to-1 correspondence between the mesh at ED phase and the mesh at ES phase by enforcing identical connectivity information and number of vertices using a radial basis function morphing approach^51^. Details of the reconstruction approach and methodology can be found in our previous publications^5,7^. The LV endocardial mesh is then partitioned into 16 segments (excluding the true apex, which cannot be easily identified on the short-axis images) based on the American Heart Association recommendation^52^ to compute all 3D regional parameters^2,5^.

### Calculation of 3D LV regional parameters

Curvedness (*C*) measures the degree of curvature at a given point, and how it deviates from flatness on a surface. It was calculated using the formula^2,16^

1 $$C=\sqrt{\frac{{k}_{1}^{2}+{k}_{2}^{2}}{2}}$$

where *k*_1_ and *k*_2_ are the maximum and minimum principal curvatures computed from the first and second fundamental form of the surface. LV wall thickness at ED (*WT*_*ED*_) and ES (*WT*_*ES*_) for each segment were calculated using a previously derived formula^2^. Thick-walled elliptical and spherical models^25,53^ had been proposed for calculating pressure-normalized wall stress, obviating the need for invasive LV pressure measurement. In the current study, the curvature-based wall stress index *σ*_*i*_, was determined from the inner radius of curvature (*R*) and wall thickness *WT*^2^:

2 $${\sigma }_{i}=\frac{R}{2WT\left(1+\frac{WT}{2R}\right)}$$

Following Grossman *et al*.^22^, peak systolic wall stress (*σ*_*ES*_) was calculated as

3 $${\sigma }_{ES}=0.133\times SP\times {\sigma }_{i,ES}$$

where *SP* denotes peak systolic ventricular blood pressure (= 0.9 × systolic blood pressure)^54^ and *σ*_*i,ES*_ is the curvature-based wall stress index computed at ES by Eq. (2). The multiplier 0.133 expressed the final result in 1000 N/m^2^.

Area strain is a quantitative strain measurement that incorporates endocardial wall strains in the circumferential, longitudinal and radial directions, and reflects the aggregate deformation of the LV endocardial surface with contraction and relaxation. The area strain (*AS*) was defined as^4^

4 $$AS=\,\mathrm{ln}\left(\frac{S{A}_{ES}}{S{A}_{ED}}\right)\times 100 \% $$

where *SA*_*ED*_ and *SA*_*ES*_ are endocardial surface areas at ED and ES phases, respectively.

### Statistical analysis

Data assembly and statistical analysis were performed with SAS version 9.4 (SAS Institute, Inc., Cary, North Carolina, USA). All continuous variables were presented as mean ± standard deviation (SD). Associations between continuous variables were investigated using regression and correlation (Pearson). Comparisons of means between two independent groups were investigated using the two-sample t test. Comparison of means in three or more groups was investigated by one-way analysis of variance (ANOVA) with Bonferroni correction. Correlations of CMR measurements with gender and age were analyzed using a two-way analysis of variance model with factors age category (≤44, 45–64, 65–74 and 75–84), gender (M/F), and age × gender interaction. F-tests were performed to test for significant differences in age category and gender main effect means, and for age × gender interaction effects.

Inter- and intra-observer reproducibility were assessed in 20 randomly chosen subjects via intraclass correlation coefficient (ICC), Bland-Altman analysis and coefficient of variation (CV). For inter-observer variability, LV endocardial and epicardial contours were segmented by a second independent observer (SL) blinded to the first observer’s results, and a second segmentation was made by the primary observer (XZ) one month after the initial segmentation to assess intra-observer variability. Statistical significance was set at *P* < 0.05.

## Supplementary information

Supplementary information.

## Data Availability

All data generated or analyzed during this study are included in this published article (and its Supplementary Information files).

## References

[1] Peng J (2018). Normal values of myocardial deformation assessed by cardiovascular magnetic resonance feature tracking in a healthy Chinese population: a multicenter study. Front. Physiol..

[2] Zhong L (2009). Left ventricular regional wall curvature and wall stress in patients with ischemic dilated cardiomyopathy. Am. J. Physiol. Heart Circ. Physiol..

[3] Zhong L (2011). Impact of surgical ventricular restoration on ventricular shape, wall stress, and function in heart failure patients. Am. J. Physiol. Heart Circ. Physiol..

[4] Teo SK, Vos FJ, Tan RS, Zhong L, Su Y (2015). Regional ejection fraction and regional area strain for left ventricular function assessment in male patients after first-time myocardial infarction. J. R. Soc. Interface.

[5] Su Y (2012). A geometrical approach for evaluating left ventricular remodeling in myocardial infarct patients. Comput. Methods Programs Biomed..

[6] Zhao X (2018). Left ventricular wall stress is sensitive marker of hypertrophic cardiomyopathy with preserved ejection fraction. Front. Physiol..

[7] Zhong L (2012). Right ventricular regional wall curvedness and area strain in patients with repaired tetralogy of Fallot. Am. J. Physiol. Heart Circ. Physiol..

[8] Chan, W. C. Singapore’s Ageing Population: Managing Healthcare And End-Of-Life Decisions. (Routledge, Oxford, UK, 2011).

[9] Leng S (2018). Age-related changes in four-dimensional CMR-derived atrioventricular junction velocities and displacements: Implications for the identification of altered annular dynamics for ventricular function assessment.. Int. J. Cardiol. Heart Vasc..

[10] Hindieh W (2017). Discrepant measurements of maximal left ventricular wall thickness between cardiac magnetic resonance imaging and echocardiography in patients with hypertrophic cardiomyopathy. Circ. Cardiovasc. Imaging.

[11] Puntmann VO (2013). Left ventricular chamber dimensions and wall thickness by cardiovascular magnetic resonance: comparison with transthoracic echocardiography. Eur. Heart J. Cardiovasc. Imaging.

[12] Kawel N (2012). Normal left ventricular myocardial thickness for middle-aged and older subjects with steady-state free precession cardiac magnetic resonance: the multi-ethnic study of atherosclerosis. Circ. Cardiovasc. Imaging.

[13] Natori S (2006). Cardiovascular function in multi-ethnic study of atherosclerosis: normal values by age, sex, and ethnicity. AJR.

[14] Zhong L (2014). Characterization and quantification of curvature using independent coordinates method in the human left ventricle by magnetic resonance imaging to identify the morphology subtype of hypertrophy cardiomyopathy. Conf. Proc. IEEE Eng. Med. Biol. Soc..

[15] Dellegrottaglie S (2007). Pulmonary hypertension: accuracy of detection with left ventricular septal-to-free wall curvature ratio measured at cardiac MR. Radiology.

[16] Koenderink JJ, Van Doorn AJ (1992). Surface shape and curvature scales. Image Vision Comput..

[17] Addetia K (2016). Three-dimensional echocardiography-based analysis of right ventricular shape in pulmonary arterial hypertension. Eur. Heart J. Cardiovasc. Imaging.

[18] Addetia K (2018). Morphologic analysis of the normal right ventricle using three-dimensional echocardiography-derived curvature indices. J. Am. Soc. Echocardiogr..

[19] Medvedofsky D (2018). 2D and 3D echocardiography-derived indices of left ventricular function and shape: relationship with mortality. JACC Cardiovasc. Imaging.

[20] Maffessanti F (2011). Three-dimensional analysis of regional left ventricular endocardial curvature from cardiac magnetic resonance images. Magn. Reson. Imaging.

[21] Badeer HS (1963). Contractile tension in the myocardium. Am. Heart J..

[22] Grossman W, Jones D, McLaurin LP (1975). Wall stress and patterns of hypertrophy in the human left ventricle. J. Clin. Invest..

[23] Yin FC (1981). Ventricular wall stress. Circ. Res..

[24] Zhong L, Ghista DN, Tan RS (2012). Left ventricular wall stress compendium. Comput. Meth. Biomech. Biomed. Eng..

[25] Alter P (2007). Relation of B-type natriuretic peptide to left ventricular wall stress as assessed by cardiac magnetic resonance imaging in patients with dilated cardiomyopathy. Can. J. Physiol. Pharmacol..

[26] Guccione JM, Costa KD, McCulloch AD (1995). Finite element stress analysis of left ventricular mechanics in the beating dog heart. J. Biomech..

[27] Lee LC (2014). Patient-specific finite element modeling of the Cardiokinetix Parachute device: effects on left ventricular wall stress and function. Med. Biol. Eng. Comput..

[28] Kleijn SA, Aly MF, Terwee CB, van Rossumm AC, Kamp O (2011). Three-dimensional speckle tracking echocardiography for automatic assessment of global and regional left ventricular function based on area strain. J. Am. Soc. Echocardiogr..

[29] Pérez de Isla L (2011). Area strain: normal values for a new parameter in healthy people. Rev. Esp. Cardiol..

[30] Taylor RJ (2015). Myocardial strain measurement with feature-tracking cardiovascular magnetic resonance: normal values. Eur. Heart J. Cardiovasc. Imaging.

[31] Augustine D (2013). Global and regional left ventricular myocardial deformation measures by magnetic resonance feature tracking in healthy volunteers: comparison with tagging and relevance of gender. J. Cardiovasc. Magn. Reson..

[32] Andre F (2015). Age- and gender-related normal left ventricular deformation assessed by cardiovascular magnetic resonance feature tracking. J. Cardiovasc. Magn. Reson..

[33] Rabbitt RD (1995). Mapping of hyperelastic deformable templates using the finite element method.. Proc. SPIE..

[34] Xi C (2016). Patient-specific computational analysis of ventricular mechanics in pulmonary arterial hypertension. J. Biomech. Eng..

[35] Finsberg H (2019). Computational quantification of patient-specific changes in ventricular dynamics associated with pulmonary hypertension. Am. J. Physiol. Heart Circ. Physiol..

[36] Zou H (2020). Three-dimensional biventricular strains in pulmonary arterial hypertension patients using hyperelastic warping. Comput. Methods Programs. Biomed..

[37] Zou H (2018). Quantification of biventricular strains in heart failure with preserved ejection fraction patient using hyperelastic warping method. Front. Physiol..

[38] Komajda M, Lam CS (2014). Heart failure with preserved ejection fraction: a clinical dilemma. Eur. Heart J..

[39] Petitjean C, Dacher JN (2011). A review of segmentation methods in short axis cardiac MR images. Med. Image Anal..

[40] Yang XL (2016). Cardiac image segmentation by random walks with dynamic shape constraint. IET Comput. Vision.

[41] Bai W (2018). Automated cardiovascular magnetic resonance image analysis with fully convolutional networks. J. Cardiovasc. Magn. Reson..

[42] Yang X, Song Q, Su Y (2017). Automatic segmentation of left ventricle cavity from short-axis cardiac magnetic resonance images. Med. Biol. Eng. Comput..

[43] Avendi MR, Kheradvar A, Jafarkhani H (2016). A combined deep-learning and deformable-model approach to fully automatic segmentation of the left ventricle in cardiac MRI. Med. Image Anal..

[44] Bernard O (2018). Deep learning techniques for automatic MRI cardiac multi-structures segmentation and diagnosis: is the problem solved?. IEEE Trans. Med. Imaging.

[45] Bizopoulos P, Koutsouris D (2019). Deep Learning in Cardiology. IEEE Rev. Biomed. Eng..

[46] Leng S (2018). Imaging 4D morphology and dynamics of mitral annulus in humans using cardiac cine MR feature tracking. Sci. Rep..

[47] Avendi MR, Kheradvar A, Jafarkhani H (2017). Automatic segmentation of the right ventricle from cardiac MRI using a learning-based approach. Magn. Reson. Med..

[48] Wang Y (2019). Fully automatic segmentation of 4D MRI for cardiac functional measurements. Med. Phys..

[49] Ho KK, Anderson KM, Kannel WB, Grossman W, Levy D (1993). Survival after the onset of congestive heart failure in Framingham heart study subjects. Circulation.

[50] Tan ML (2013). A geometrical approach for automatic shape restoration of the left ventricle. PloS One.

[51] Su, Y., Teo, S. K., Lim, C. W., Zhong, L. & Tan, R. S. Automatic generation of endocardial surface meshes with 1-to-1 correspondence from cine-MR images. Proceedings of SPIE 9414, Medical Imaging 2015: Computer-Aided Diagnosis, 941431, Orlando, FL (2015).

[52] Cerqueira MD (2002). Standardized myocardial segmentation and nomenclature for tomographic imaging of the heart: a statement for healthcare professional from the Cardiology of the American Heart Association. Circulation.

[53] Zhong L (2006). Left ventricular shape-based contractility index. J. Biomech..

[54] Reichek N (1982). Noninvasive determination of left ventricular end-systolic stress: validation of the method and initial application. Circulation.

