# Supplementary material for: Reference Ranges for Left Ventricular Curvedness and Curvedness-Based Functional Indices Using Cardiovascular Magnetic Resonance in Healthy Asian Subjects
Source: Sci Rep. 2020 May 21;10:8465. doi: 10.1038/s41598-020-65153-3 (PMC7242400; doi:10.1038/s41598-020-65153-3)
Supplement: Supplementary file 1 — Supplementary information. [file 41598_2020_65153_MOESM1_ESM.docx]

**Reference Ranges for Left Ventricular Curvedness and Curvedness-Based Functional Indices Using Cardiovascular Magnetic Resonance in Healthy Asian Subjects**

Xiaodan Zhao^1#^, Soo-Kng Teo^2#^, Liang Zhong^1,3*$^, Shuang Leng^1^, Jun-Mei Zhang^1,3^, Ris Low^1^, John Allen^3^, Angela S. Koh^1,3^, Yi Su^2$^, Ru-San Tan^1,3$^

^1^National Heart Research Institute Singapore, National Heart Centre Singapore, 5 Hospital Drive, Singapore, 169609, Singapore

^2^Institute of High Performance Computing, Agency for Science, Technology and Research (A*STAR), 1 Fusionopolis Way, #16-16 Connexis, Singapore 138632, Singapore

^3^Duke-NUS Medical School, 8 College Road, Singapore, 169857, Singapore

*Correspondence: Liang Zhong, [zhong.liang@nhcs.com.sg](mailto:zhong.liang@nhcs.com.sg)

Address: National Heart Center Singapore, 5 Hospital Drive, 169609, Singapore.

^#^Joint first author

^$^Joint last author

Appendix Table 1. Baseline demographics and left ventricular parameters for subject groups.

| **Parameters** | **Total**  **(n = 171)** | **20–29**  **(n = 35)** | **30–39**  **(n = 20)** | **40–49**  **(n = 22)** | **50–59**  **(n = 15)** | **60–69**  **(n = 34)** | **≥70**  **(n = 45)** | ***P* Value** |
| --- | --- | --- | --- | --- | --- | --- | --- | --- |
| Male, n (%) | 83 (48.5%) | 17 (48.6%) | 9 (45.0%) | 9 (40.9%) | 6 (40.0%) | 16 (47.1%) | 26 (57.8%) | 0.744 |
| Age, years (range) | 52 ± 19 | 25 ± 3 | 34 ± 3 | 45 ± 2 | 55 ± 3 | 65 ± 3 | 73 ± 2 | <0.001 |
| Height, cm | 163 ± 9 | 166 ± 10 | 164 ± 9 | 166 ± 9 | 160 ± 9 | 162 ± 7 | 160 ± 7* | 0.013 |
| Weight, kg | 61 ± 12 | 62 ± 17 | 62 ± 12 | 69 ± 15 | 60 ± 10 | 60 ± 9 | 58 ± 9^$^ | 0.011 |
| Body surface area, m^2^ | 1.66 ± 0.20 | 1.68 ± 0.27 | 1.68 ± 0.20 | 1.78 ± 0.23 | 1.64 ± 0.17 | 1.64 ± 0.14 | 1.60 ± 0.14 | 0.011 |
| Body mass index, kg/m^2^ | 23.0 ± 3.3 | 22.3 ± 3.7 | 23.1 ± 2.8 | 25.1 ± 3.6 | 23.6 ± 2.5 | 22.6 ± 2.9 | 22.5 ± 3.1^$^ | 0.049 |
| SBP, mmHg | 135 ± 20 | 125 ± 13 | 128 ± 19 | 128 ± 16 | 128 ± 13 | 137 ± 19 | 150 ± 21*^#$^§^ | <0.001 |
| DBP, mmHg | 77 ± 10 | 74 ± 11 | 77 ± 11 | 76 ± 10 | 79 ± 7 | 77 ± 9 | 79 ± 10 | 0.506 |
| Heart rate, bpm | 76 ± 12 | 79 ± 11 | 77 ± 13 | 74 ± 12 | 77 ± 15 | 77 ± 15 | 76 ± 12 | 0.883 |
| LV EDV index, ml/m^2^ | 69 ± 11 | 73 ± 9 | 75 ± 10 | 73 ± 11 | 74 ± 10 | 68 ± 11 | 61 ± 10*^#$^^ | <0.001 |
| LV ESV index, ml/m^2^ | 25 ± 7 | 29 ± 6 | 27 ± 7 | 27 ± 7 | 24 ± 5 | 26 ± 6 | 21 ± 6*^#$§^ | <0.001 |
| LV SV index, ml/m^2^ | 44 ± 7 | 44 ± 5 | 47 ± 7 | 46 ± 7 | 50 ± 8 | 42 ± 7^^^ | 41 ± 7^#$^^ | <0.001 |
| LV ejection fraction, % | 64 ± 6 | 61 ± 5 | 64 ± 7* | 64 ± 6 | 64 ± 6 | 62 ± 5 | 67 ± 7^§^ | 0.001 |
| LV mass index, g/m^2^ | 45 ± 11 | 44 ± 13 | 46 ± 9 | 46 ± 11 | 51 ± 8 | 43 ± 8 | 45 ± 12 | 0.367 |
| LV mass-to-volume ratio, g/ml | 0.65 ± 0.16 | 0.60 ± 0.16 | 0.63 ± 0.15 | 0.63 ± 0.15 | 0.65 ± 0.13 | 0.64 ± 0.13 | 0.73 ± 0.16* | 0.005 |
| ED curvedness, mm^-1^ | 0.041 ± 0.004 | 0.041 ± 0.005 | 0.040 ± 0.003 | 0.040 ± 0.004 | 0.040 ± 0.005 | 0.040 ± 0.004 | 0.042 ± 0.004 | 0.206 |
| ES curvedness, mm^-1^ | 0.068 ± 0.011 | 0.066 ± 0.011 | 0.066 ± 0.007 | 0.069 ± 0.011 | 0.066 ± 0.010 | 0.066 ± 0.010 | 0.073 ± 0.012* | 0.010 |
| ED wall thickness, mm | 4.92 ± 0.77 | 4.77 ± 0.88 | 4.80 ± 0.72 | 4.93 ± 0.76 | 5.00 ± 0.54 | 4.81 ± 0.59 | 5.14 ± 0.86 | 0.357 |
| ES wall thickness, mm | 7.75 ± 1.22 | 7.29 ± 1.17 | 7.55 ± 1.26 | 7.80 ± 1.13 | 7.92 ± 0.91 | 7.64 ± 0.86 | 8.21 ± 1.48* | 0.057 |
| ED wall stress index | 2.72 ± 0.46 | 2.82 ± 0.45 | 2.85 ± 0.45 | 2.79 ± 0.54 | 2.67 ± 0.35 | 2.79 ± 0.44 | 2.53 ± 0.44 | 0.041 |
| ES wall stress index | 0.99 ± 0.24 | 1.10 ± 0.19 | 1.05 ± 0.26 | 1.00 ± 0.25 | 0.95 ± 0.18 | 1.00 ± 0.21 | 0.86 ± 0.24*^#^ | <0.001 |
| Peak systolic wall stress, 1000 N/m^2^ | 15.7 ± 3.6 | 16.3 ± 3.2 | 15.8 ± 3.2 | 15.2 ± 3.8 | 13.8 ± 2.3 | 16.5 ± 3.5 | 15.2 ± 4.0 | 0.251 |
| Area strain, % | 69 ± 11 | 63 ± 9 | 68 ± 9 | 72 ± 10 | 69 ± 10 | 67 ± 10 | 75 ± 12^§^ | <0.001 |

Data were represented as mean ± SD or percentage. SBP: systolic blood pressure; DBP: diastolic blood pressure; LV: left ventricle; ED: end-diastolic; ES: end-systolic; EDV: end-diastolic volume; ESV: end-systolic volume. *P* values are from one-way analysis of variance (ANOVA) across the six age groups with Bonferroni test, a *P* values of 0.05 was considered significant. *Significant difference compared with group 20-29; ^#^significant difference compared with group 30-39; ^$^significant difference compared with group 40-49; ^^^significant difference compared with group 50-59; ^§^significant difference compared with group 60-69.
